# Investigating the technical feasibility of magnetoencephalography during transcranial direct current stimulation

**DOI:** 10.3389/fnhum.2023.1270605

**Published:** 2023-09-13

**Authors:** Yuichiro Shirota, Motofumi Fushimi, Masaki Sekino, Masato Yumoto

**Affiliations:** ^1^Department of Clinical Laboratory, The University of Tokyo Hospital, Tokyo, Japan; ^2^Department of Bioengineering, The Graduate School of Engineering, The University of Tokyo, Tokyo, Japan; ^3^Department of Clinical Engineering, Gunma Paz University, Takasaki, Japan

**Keywords:** MEG, tDCS, artifact rejection, magnetoencephalography, Maxwell filter, source modeling, transcranial direct current stimulation

## Abstract

**Introduction:**

Magnetoencephalography (MEG) can measure weak magnetic fields produced by electrical brain activity. Transcranial direct current stimulation (tDCS) can affect such brain activities. The concurrent application of both, however, is challenging because tDCS presents artifacts on the MEG signal. If brain activity during tDCS can be elucidated by MEG, mechanisms of plasticity-inducing and other effects of tDCS would be more comprehensively understood. We tested the technical feasibility of MEG during tDCS using a phantom that produces an artificial current dipole simulating focal brain activity. An earlier study investigated estimation of a single oscillating phantom dipole during tDCS, and we systematically tested multiple dipole locations with a different MEG device.

**Methods:**

A phantom provided by the manufacturer was used to produce current dipoles from 32 locations. For the 32 dipoles, MEG was recorded with and without tDCS. Temporally extended signal space separation (tSSS) was applied for artifact rejection. Current dipole sources were estimated as equivalent current dipoles (ECDs). The ECD modeling quality was assessed using localization error, amplitude error, and goodness of fit (GOF). The ECD modeling performance with and without tDCS, and with and without tSSS was assessed.

**Results:**

Mean localization errors of the 32 dipoles were 1.70 ± 0.72 mm (tDCS off, tSSS off, mean ± standard deviation), 6.13 ± 3.32 mm (tDCS on, tSSS off), 1.78 ± 0.83 mm (tDCS off, tSSS on), and 5.73 ± 1.60 mm (tDCS on, tSSS on). Mean GOF findings were, respectively, 92.3, 87.4, 97.5, and 96.7%. Modeling was affected by tDCS and restored by tSSS, but improvement of the localization error was marginal, even with tSSS. Also, the quality was dependent on the dipole location.

**Discussion:**

Concurrent tDCS-MEG recording is feasible, especially when tSSS is applied for artifact rejection and when the assumed location of the source of activity is favorable for modeling. More technical studies must be conducted to confirm its feasibility with different source modeling methods and stimulation protocols. Recovery of single-trial activity under tDCS warrants further research.

## 1. Introduction

In recent years, non-invasive brain stimulation techniques such as transcranial direct current stimulation (tDCS) have attracted attention for their potential to modulate brain activity. tDCS involves the application of weak electrical currents through scalp electrodes to modify the excitability of underlying cortical regions (Nitsche et al., 2008; Antal et al., 2017). It offers promising prospects for both basic research and clinical applications ranging from neurorehabilitation to the treatment of neurological and psychiatric disorders (Lefaucheur et al., 2017). To elucidate the underlying neural mechanisms and to optimize stimulation protocols, it is necessary to investigate the effects of tDCS in terms of brain activity and dynamics. One powerful neurophysiological technique that can provide valuable insights in this context is magnetoencephalography (MEG).

MEG is a non-invasive method that measures the weak magnetic fields generated by neuronal activity in the brain. It offers excellent temporal resolution with millisecond-level precision, enabling the assessment of rapid changes in neural dynamics (Hari et al., 2018). By capturing the spatiotemporal patterns of electrical activity in the brain, MEG can provide unique opportunities to investigate tDCS effects on the brain’s oscillatory activity, functional connectivity, and network dynamics.

Integrating MEG with tDCS, however, presents technical challenges because of the potential interference caused by electrical currents generated during tDCS. If brain activity during tDCS can be elucidated by MEG, mechanisms of plasticity-inducing and other effects of tDCS would be more comprehensively understood. Earlier studies have made notable strides in overcoming these technical challenges and in combining MEG with tDCS successfully. Soekadar et al. demonstrated the feasibility of using MEG to assess human brain oscillations during the application of transcranial electric currents (Soekadar et al., 2013). Their study highlighted the potential of MEG to characterize tDCS effects on cortical activity and provided insights into tDCS modulatory effects on brain oscillations. They reported that estimation error was 2.23–4.24 mm without tDCS and 2.24–11.18 mm with tDCS by repeated measurements of a single dipole. Building upon this foundation, Garcia-Cossio et al. conducted simultaneous tDCS and whole-head MEG recordings to assess tDCS effects on slow cortical magnetic fields (Garcia-Cossio et al., 2016). Their work not only demonstrated the feasibility of combining tDCS and MEG; it also provided valuable insights into the effects of tDCS on cortical dynamics. These studies advanced our understanding of the modulatory effects of tDCS on brain oscillations and provided evidence for the utility of MEG in investigating tDCS-induced changes in cortical activity.

Whereas these studies have demonstrated the technical feasibility of combining MEG with tDCS, further advancements must be achieved to exploit the potential of this multimodal approach fully, including development of advanced artifact removal techniques specific to tDCS-MEG recordings. As described herein, we aimed at exploring the technical feasibility of conducting MEG recordings during tDCS through phantom models. More specifically, we conducted a phantom study with and without tDCS, and investigated the efficiency of an artifact rejection technique designated as temporally extended signal space separation (tSSS). The theory of tSSS is based on Maxwell’s equations. Actually, tSSS can distinguish signals from inside a sphere containing sources of the measured magnetic field, and signals from outside of it (Taulu et al., 2005; Taulu and Simola, 2006). Importantly, nearby artifacts such as those originating from tDCS are expected to be difficult to remove using signal source separation (SSS) alone, but tSSS is expected to be more suitable by considering temporal correlation between the two signal spaces (Taulu and Simola, 2006). We compared the quality of source modeling between tDCS-off and tDCS-on, and that between tSSS-off and tSSS-on. To expand evidence shown by Soekadar et al. (2013), we tested 32 different dipole locations.

## 2. Materials and methods

### 2.1. Phantom

A phantom provided by the MEG device manufacturer (MEGIN OY, Espoo, Finland) was used to measure weak magnetic fields under the presence of tDCS. It contains 32 different artificial dipoles in different locations (Supplementary Figure S1). Some dipoles are located superficially and others more deeply, enabling us to test the influence of depth of the dipole. In general, deep MEG sources are more difficult to locate because magnetic fields from such dipoles attenuate according to the distance to the sensors. The amplitude of the dipoles was set as 100 nAm, which is comparable to sources deriving from human brain activity. The correct dipole locations are presented as a Supplementary Table S1.

### 2.2. Transcranial direct current stimulation

**Figure 1:**
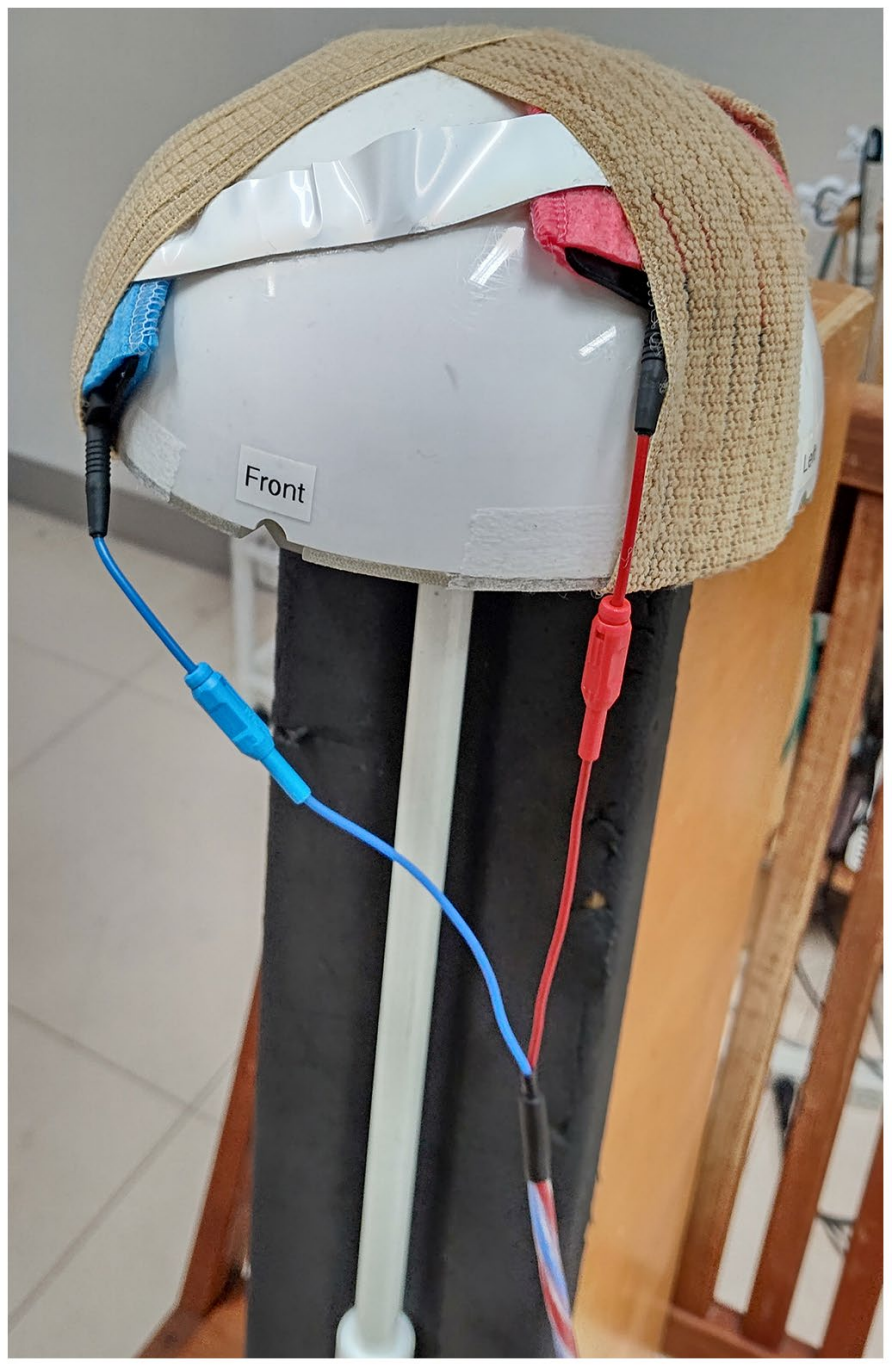
Setup of the phantom-tDCS experiments. Electrodes for transcranial direct current stimulation (tDCS) were fixed over the MEG phantom with rubber bands: the red electrode is anodal; the blue electrode is cathodal. The white band between the electrodes is a gel electrode providing a path for the electric current.

Using a DC stimulator (DC-Stimulator MR; Neurocare Group AG, Munich, Germany) with an anode and a cathode, each with a rectangular shape of 5 × 7 cm, tDCS was applied. The electrodes were covered with a sponge soaked in saline solution. The anode was placed on the left convex; the cathode was placed over the right frontal edge of the phantom, resembling anodal stimulation of the left primary motor cortex with the cathodal “return” electrode over the right supraorbital area. This montage has been used frequently for human experiments (Nitsche and Paulus, 2000, 2001). To simulate a superficial current path, a gel-electrode band (GE HealthCare, Chicago, Illinois, United States) was placed to connect the two electrodes. Figure 1 portrays these settings. The stimulus intensity was set at 1 mA, and fluctuation of the currents was up to 0.01 mA.

### 2.3. Measurements

Using a VectorView device (MEGIN OY, Espoo, Finland), which has 204 planar gradiometers and 102 magnetometers, MEG recordings were performed with the sampling frequency of 600 Hz, and with a band-pass filter for 0.10–200 Hz. Four Head Position Indicator (HPI) coils in the phantom were digitized using a tracker (Polhemus Inc., Colchester, Vermont, United States); then the phantom was situated in the MEG sensor helmet. Conditions with and without tDCS were tested separately. In measurements with tDCS, stimulation was turned on after MEG recording was started; starting tDCS disturbed the recording, so that channels had to be reset after beginning tDCS. Then, the phantom dipole was activated to collect 200 responses. Each of the 32 dipoles was obtained sequentially in the same manner. Measurement without tDCS did not need channel-resetting, but was otherwise conducted similarly.

### 2.4. Data analysis

**Figure 2:**
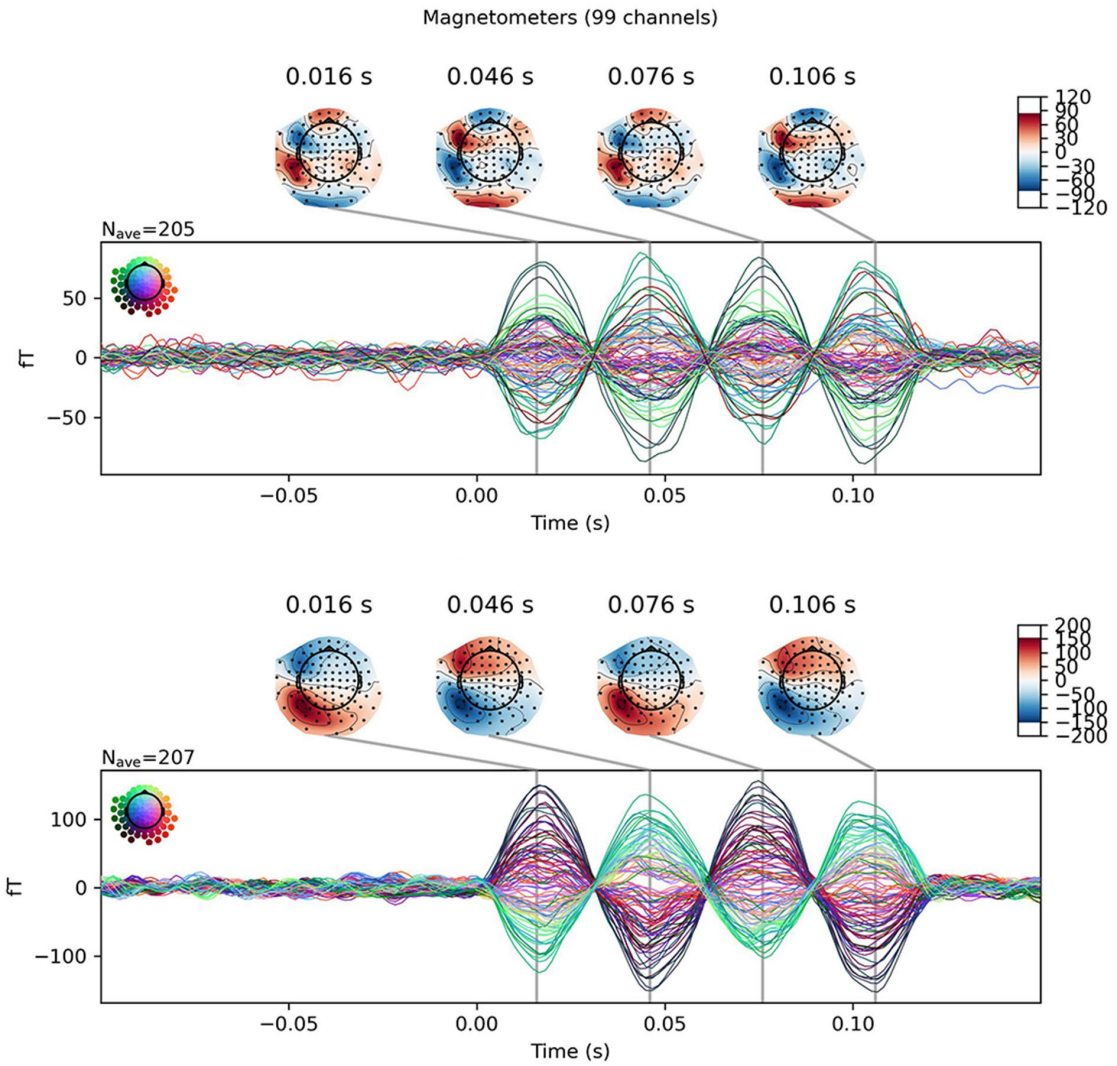
Example for sensor level data of a single dipole. Averaged results from dipole #20 are presented as butterfly plots. Isofield maps are shown above the peaks: Top, without tSSS; Bottom, with tSSS. The differences in the scale and distributions exhibited in the isofield maps is noteworthy.

Equivalent current dipoles (ECDs) were estimated from averaged signals: results of the first trial were discarded because they possibly contained some artifact caused by dipole switching. All 306 channels were included to solve the inverse problem using the MNE Python package (version 1.3^1^; Gramfort et al., 2013). As part of artifact rejection, tSSS was performed using proprietary software (MEGIN OY, Espoo, Finland). The origin was set at (0, 0, 0) with the device coordinate. The buffer length and correlation limit were, respectively, 10 s and 0.98. After the signal was band-pass filtered between 1 and 100 Hz and a notch filter at 50 Hz was applied, ECD was fit at the second peak of the artificial dipoles at 46 ms (Figure 2).

As a measure of quality of ECD fitting, the localization error, amplitude error, and goodness of fit (GOF) were calculated. The correct values for dipole location were obtained using the mne.dipole.get_phantom_dipoles() function of the MNE Python (version 1.3 (see footnote 1); Gramfort et al., 2013). The localization error was defined as the Euclidean distance between the estimated and correct dipole location. The correct amplitude was set at 50 nAm: half of the peak-to-peak amplitude of 100 nAm. The estimated amplitude was subtracted from it. Similarly to the coefficient of determination, GOF denotes how much of the variance is explainable by the estimated ECD. Repeated-measures two-way analysis of variance (ANOVA) was conducted with two within-subjects factors of tDCS and tSSS, including the presence or absence of them. Each dipole location served as a subject. For statistical analysis, the amplitude difference was represented as absolute values rather than as raw subtracted values. Data processing and statistical analyses were performed using MNE Python (ver. 1.3 (see footnote 1); Gramfort et al., 2013) and other Python libraries including SciPy.^4^ Significance was inferred for results with *p* < 0.05.

## 3. Results

**Figure 3:**
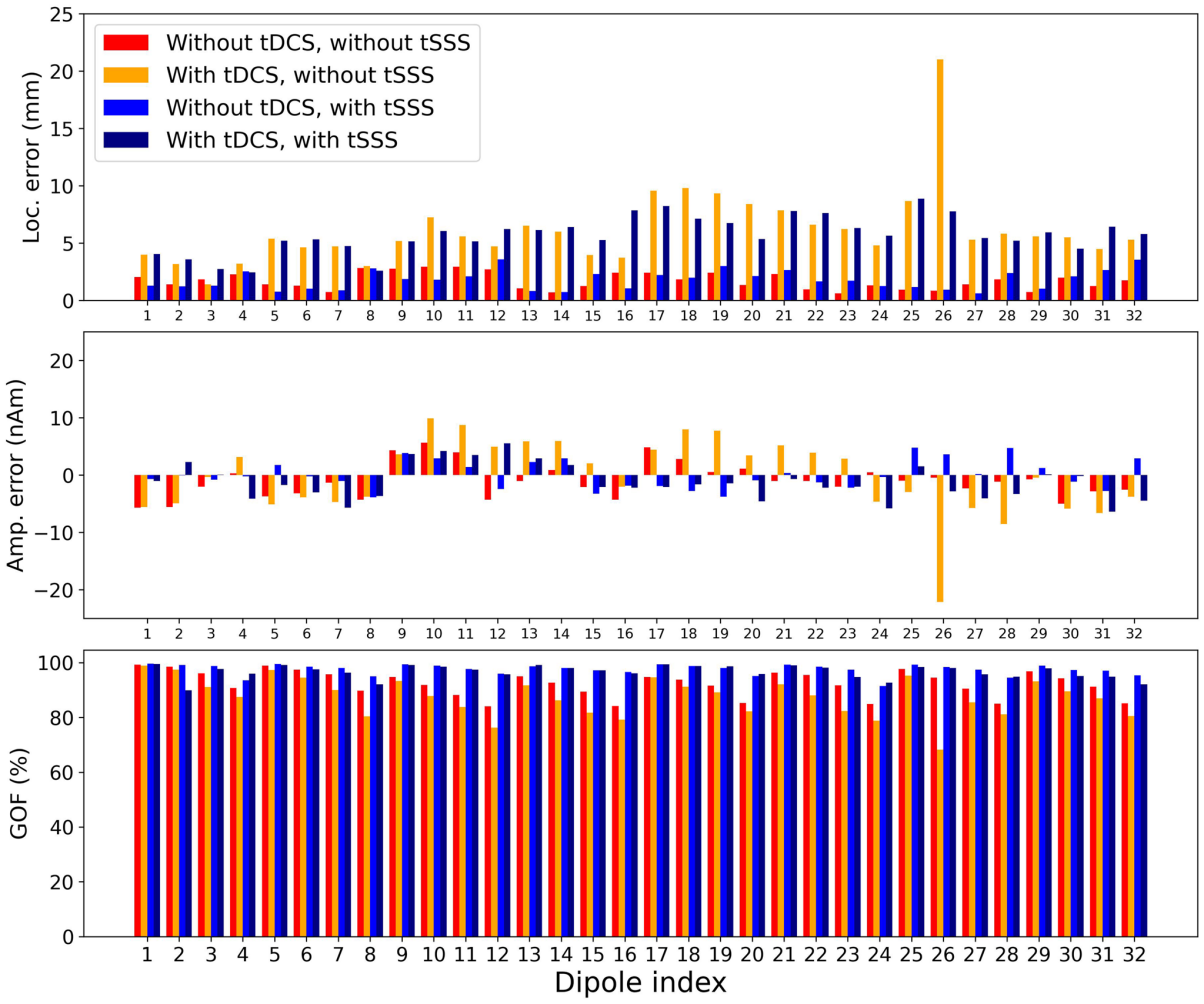
Quality of modeling for data. Localization error, amplitude error, and goodness of fit (GOF) are demonstrated for the 46 ms peak of each dipole. The x-axis shows the dipole index. The y-axis shows each measure. Each bar represents from left to right: without tDCS and without tSSS (red), with tDCS and without tSSS (orange), without tDCS and with tSSS (blue), and with tDCS and with tSSS (navy). The with-tDCS condition performs worse.

All quality parameters, i.e., localization error, amplitude error, and GOF, were better with measurement without tDCS (Figure 3). Artifact rejection with tSSS improved the quality, but the localization error was, on average, comparable (Table 1). Two-way repeated-measures ANOVA revealed that the main factor tDCS and interaction between tDCS and tSSS were significant for all parameters except for the interaction for the localization error. The main factor tSSS was significant for the absolute amplitude error and GOF (Table 2).

**Table 1:** Quality parameters of ECD modeling.

	Localization error	Amplitude error	GOF
tDCS off/tSSS off	1.70 ± 0.72 mm	2.6 ± 1.8 nAm	92.3 ± 4.66%
tDCS on/tSSS off	6.13 ± 3.32 mm	5.4 ± 4.0 nAm	87.4 ± 6.98%
tDCS off/tSSS on	1.78 ± 0.83 mm	2.0 ± 1.4 nAm	97.5 ± 1.94%
tDCS on/tSSS on	5.73 ± 1.60 mm	2.9 ± 1.7 nAm	96.7 ± 2.44%

ECD, equivalent current dipole; GOF, goodness of fit; tDCS, transcranial direct current stimulation; tSSS, temporally extended signal space separation.

**Table 2:** Results of repeated-measures ANOVA.

	*F*	df	*p*
Localization error			
tDCS	94.6	1, 31	<0.001
tSSS	0.42	1, 31	0.52
Interaction	0.96	1, 31	0.33
Amplitude error (absolute value)			
tDCS	18.5	1, 31	<0.001
tSSS	16.4	1, 31	<0.001
Interaction	4.50	1, 31	0.041
GOF			
tDCS	45.7	1, 31	<0.001
tSSS	81.6	1, 31	<0.001
Interaction	22.4	1, 31	<0.001

ANOVA, analysis of variance; df, degree of freedom; GOF, goodness of fit; tDCS, transcranial direct current stimulation; tSSS, temporally extended signal space separation.

*Post-hoc* analyses were performed on each tSSS level. Without tSSS, the localization error and absolute amplitude error were significantly larger and GOF was significantly smaller with tDCS than without [Table 1, *t*(31) = 4.47, *p* < 0.001, *t*(31) = 3.52, *p* = 0.001, and *t*(31) = −6.26, *p* < 0.001 by paired *t-*test, respectively]. The situation was similar when tSSS was applied, with numerically larger *p-*values [*t*(31) = 2.99, *p* = 0.005 for the localization error, *t*(31) = 2.19, *p* = 0.04 for the absolute amplitude error, and *t*(31) = −2.41, *p* = 0.02 for GOF].

**Figure 4:**
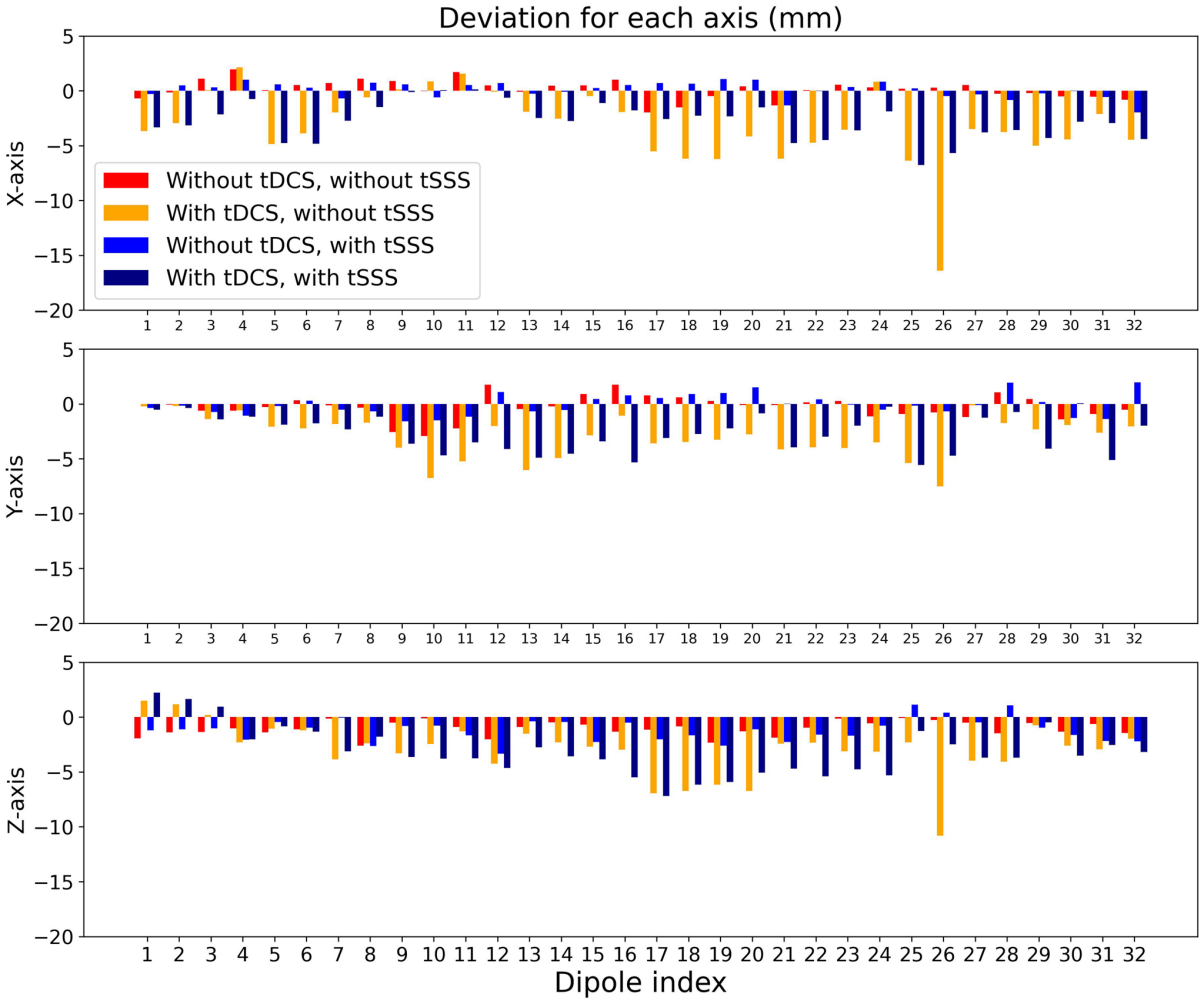
Directional biases of localization error. Localization errors are presented for X-, Y-, and Z-axis separately for each dipole. The arrangements of the bars are the same as in Figure 3: without tDCS and without tSSS (red), with tDCS and without tSSS (orange), without tDCS and with tSSS (blue), and with tDCS and with tSSS (navy).

We further investigated directional biases by subtracting actual X, Y, and Z coordinates of each dipole from estimated ones. As shown in Figure 4, the degree of deviations was comparable across the X-, Y-, and Z-axes. It is of note that deviations to the left (negative X), posterior (negative Y), and inferior (negative Z) was predominant especially with tDCS.

From looking into additional details of the modeling quality in terms of the dipole locations, three findings are noteworthy. First, the localization errors were consistently larger with tDCS for dipoles #17 to #20, especially without tSSS. These dipoles were located in the left hemisphere of the phantom, just above which the anode of the tDCS was placed. Second, the GOF for dipoles #4, #8, #12, #16, #20, #24, #28, and #32 were lower without tSSS, but the GOF was larger after tSSS. These dipoles were located deeper. Such dipoles might have been more susceptible to tDCS, but tSSS improved the situation considerably. Finally, the localization errors for dipoles #29 to #32 were not improved by tSSS, but rather larger after tSSS. These dipoles were situated in the frontal part, where MEG sensors were sparse to maintain the sight of participants.

## 4. Discussion

The findings have demonstrated that concurrent application of tDCS strongly affects ECD fitting of the MEG data, but that artifact rejection by tSSS can restore the signal quality. Our investigation consisted of systematic phantom testing, thereby providing basic data and benchmarking for future human recording.

Without tSSS, the localization error was greater with dipoles in the left hemisphere. Moreover, GOF was lower with deeper dipoles in the presence of tDCS. It is particularly interesting that the former was improved only slightly by tSSS, whereas the latter was restored to a considerable degree by tSSS. In the current experiment, the tDCS electrode was on the left hemisphere (anode) and right frontal edge (cathode) of the phantom, probably producing greater noise in the left hemisphere. This observation implies that MEG signals from brain areas immediately under the tDCS might be difficult to restore, even with tSSS. Further artifact rejection techniques might be necessary to investigate such brain areas, but activities distant from the tDCS electrodes can be estimated reliably when assisted by tSSS. Also, GOF was smaller for deeper dipoles than for superficial ones, even without tDCS. This finding is presumably attributable to the attenuation of deep signals according to the distance between the sensors and the source, decreasing the signal-to-noise ratio of such dipoles. Under these circumstances, suppression of environmental noise with tSSS would increase the GOF. Both with and without tDCS, tSSS was effective in this regard. The directional bias to the left (negative X), posterior (negative Y), and inferior (negative Z) during tDCS was intriguing, but was not totally consistent with the tDCS current flow that was left-to-right, posterior-to-anterior, and superior-to-inferior (from the red anode to the blue cathode in Figure 1).

The findings indicate tSSS as a good way to reject artifacts derived from tDCS. Compared to SSS, tSSS is expected to be better at removing artifacts near the head. By considering temporal correlations between signals inside and outside a sphere lying approximately around the MEG sensor array, tSSS can project out nearby sources (Taulu et al., 2005). The noise from tDCS is expected to pave the way through the skin along the shortest path between the anode and cathode, thereby comprising the very type of artifact that is presumed to be rejected by tSSS, but not by SSS.

Estimation errors for frontal dipoles were worse even without tDCS. These worse errors are probably attributable to insufficient MEG sensor coverage such that the sensor helmet does not cover the eyes of a subject. Activity from these parts of the brain is presumably vulnerable to variable noise; tSSS alone might be unable to recover the true signals.

Most human studies testing concurrent tDCS-MEG deal with oscillatory activity of the brain. In such studies, the power of different frequency bands was defined as the outcome of concurrent tDCS (Hanley et al., 2016; Marshall et al., 2016; Wilson et al., 2018), where source analysis was performed using different spatial filter methods. We addressed the issue of measuring short-lived evoked fields using ECD modeling, which is reasonable for estimating the sources of single, local, and prominent current activity. Our results are expected to be applicable for the source modeling used in the literature.

Furthermore, investigations into transcranial alternating current stimulation (tACS) have reportedly benefited from the integration of MEG. The potential of MEG was emphasized as a tool to uncover brain dynamics during tACS (Neuling et al., 2015). Their study highlighted the synergistic relation between MEG and tACS for elucidating the effects of this stimulation modality on brain oscillations. Their study also provided insights into the neural mechanisms underlying tACS-induced modulations. Because artifact rejection of MEG during tACS is expected to be more difficult as a result of more complicated time-course of artifacts from the stimulation, additional studies must be conducted to test its feasibility for evoked fields and other measures.

Our study has several limitations. First, we did not test an actual human brain. Human studies should incorporate more noise including physiological noises. Artifact rejection might be more complicated. Second, no current from tDCS entered into the phantom in the current investigation, precluding more complex interaction between the dipoles and interfering currents. The quality of source modeling used for this study, especially in terms of dipole locations, will nevertheless serve as a good benchmark for future human studies. Additionally, we did not test different montages of tDCS. Systematic investigations for different electrode montages are promising future directions. Testing the utility of tSSS with more complicated tDCS including high-definition tDCS (Iordan et al., 2022), for which more than two electrodes are placed, is another. For dipoles with larger errors including #25 and #26, more background noises were noted in the raw signal for unknown reasons. Since we did not measure each dipole multiple times as in Soekadar et al. (2013), such consideration may be needed for further research. Last but not least, we did not assess recovery of the signal at the single-trial level. Since non-invasive brain stimulations are more and more used in a closed-loop manner (Nasr et al., 2022), performance of artifact rejection at this level needs to be further investigated.

In conclusion, we demonstrated that tDCS affects MEG signals, but also that artifact rejection techniques such as tSSS would restore them at least partially. Human studies of MEG during tDCS are expected to shed light on the causal influence of a certain brain area on others. Results of this study are expected to be informative to plan such human studies.

## Data availability statement

The datasets presented in this article are not readily available because each request must be judged to ascertain whether it is reasonable. Requests to access the datasets should be directed to the corresponding author.

## Author contributions

YS: Conceptualization, Data curation, Formal analysis, Funding acquisition, Investigation, Methodology, Resources, Visualization, Writing – original draft. MF: Methodology, Software, Validation, Writing – review & editing. MS: Funding acquisition, Methodology, Supervision, Validation, Writing – review & editing. MY: Conceptualization, Methodology, Resources, Supervision, Validation, Writing – review & editing.

## Funding

This work was supported by JSPS KAKENHI (Grant No. JP 21 K07454) to YS and MEXT Quantum Leap Flagship Program (MEXT Q-LEAP) (Grant Nos. JPMXS0118067395 and JPMXS0118068379) to MS.

## Conflict of interest

The author(s) declared that they were an editorial board member of Frontiers, at the time of submission. This had no impact on the peer review process and the final decision.

## Publisher’s note

All claims expressed in this article are solely those of the authors and do not necessarily represent those of their affiliated organizations, or those of the publisher, the editors and the reviewers. Any product that may be evaluated in this article, or claim that may be made by its manufacturer, is not guaranteed or endorsed by the publisher.

## Footnotes

^1^ https://mne.tools/stable/index.html

^4^ https://scipy.org/
