# Supplementary material for: Investigating the technical feasibility of magnetoencephalography during transcranial direct current stimulation
Source: Front Hum Neurosci. 2023 Sep 13;17:1270605. doi: 10.3389/fnhum.2023.1270605 (PMC10525331; doi:10.3389/fnhum.2023.1270605)
Supplement: Supplementary file 1 [file Data_Sheet_1.docx]

Supplementary Material

Investigating technical feasibility of magnetoencephalography during transcranial direct current stimulation

Yuichiro Shirota^*^, Motofumi Fushimi, Masaki Sekino, Masato Yumoto

*** Correspondence:** Yuichiro Shirota: [yshirota@m.u-tokyo.ac.jp](about:blank)

# Supplementary Figures and Tables

## Supplementary Figure Coordinate system and phantom dipole locations
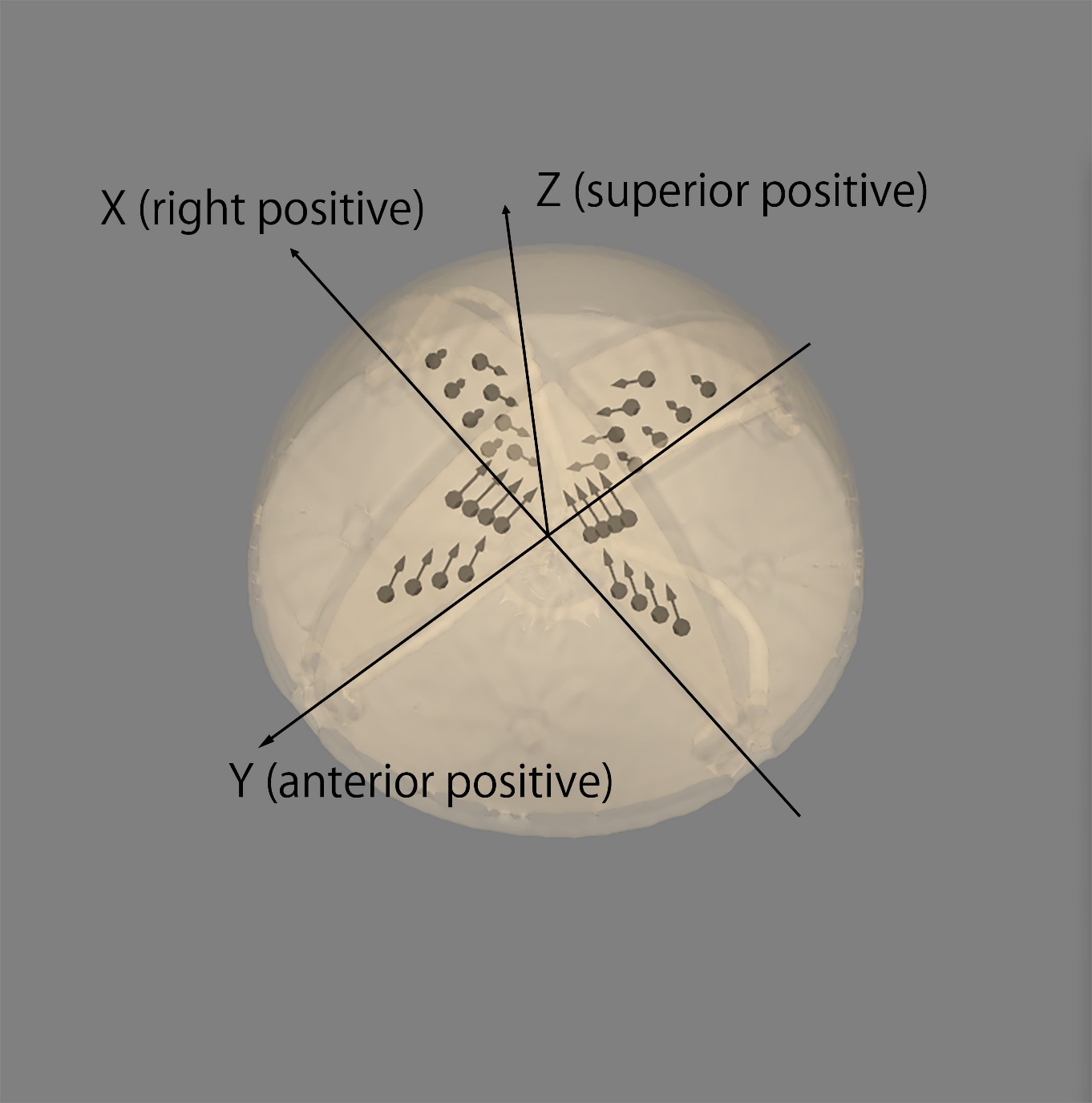


The beige dome represents the phantom surface. This figure presents the view from the upper left-front. The phantom and MEG coordinate system uses the RAS system: the x-axis is from left to right; the y-axis is from posterior to anterior; and the z-axis is from inferior to superior. The phantom dipoles are arranged on the xz-plane and yz-plane, as shown by the dots with an arrow for each. The figure was created with MNE-Python (ver. 1.3, [https://mne.tools/stable/index.html](about:blank); Gramfort et al., 2013).

## Supplementary Table. Coordinates for each dipole

| Dipole No. | x (mm) | y (mm) | z (mm) |
| --- | --- | --- | --- |
| 1 | 56.30 | 0.00 | 32.50 |
| 2 | 47.60 | 0.00 | 27.50 |
| 3 | 39.00 | 0.00 | 22.50 |
| 4 | 30.30 | 0.00 | 17.50 |
| 5 | 32.50 | 0.00 | 56.30 |
| 6 | 27.50 | 0.00 | 47.60 |
| 7 | 22.50 | 0.00 | 39.00 |
| 8 | 17.50 | 0.00 | 30.30 |
| 9 | 0.00 | -56.30 | 32.50 |
| 10 | 0.00 | -47.60 | 27.50 |
| 11 | 0.00 | -39.00 | 22.50 |
| 12 | 0.00 | -30.30 | 17.50 |
| 13 | 0.00 | -32.50 | 56.30 |
| 14 | 0.00 | -27.50 | 47.60 |
| 15 | 0.00 | -22.50 | 39.00 |
| 16 | 0.00 | -17.50 | 30.30 |
| 17 | -56.30 | 0.00 | 32.50 |
| 18 | -47.60 | 0.00 | 27.50 |
| 19 | -39.00 | 0.00 | 22.50 |
| 20 | -30.30 | 0.00 | 17.50 |
| 21 | -32.50 | 0.00 | 56.30 |
| 22 | -27.50 | 0.00 | 47.60 |
| 23 | -22.50 | 0.00 | 39.00 |
| 24 | -17.50 | 0.00 | 30.30 |
| 25 | 0.00 | 32.50 | 56.30 |
| 26 | 0.00 | 27.50 | 47.60 |
| 27 | 0.00 | 22.50 | 39.00 |
| 28 | 0.00 | 17.50 | 30.30 |
| 29 | 0.00 | 56.30 | 32.50 |
| 30 | 0.00 | 47.60 | 27.50 |
| 31 | 0.00 | 39.00 | 22.50 |
| 32 | 0.00 | 30.30 | 17.50 |
